# From molecular damage to regulatory constraint: epigenetic and metabolic limits of cellular plasticity in aging

**DOI:** 10.3389/fragi.2026.1872180

**Published:** 2026-06-26

**Authors:** Antoni R. Godlewski, Tomasz Dziaman

**Affiliations:** Department of Clinical Biochemistry, Collegium Medicum in Bydgoszcz, Nicolaus Copernicus University in Toruń, Bydgoszcz, Poland

**Keywords:** aging, epigenetic aging, epigenetic plasticity, metabolism, TET dioxygenases, Wnt signaling, cellular senescence, regulatory networks

## Abstract

Aging is most often portrayed as the progressive buildup of molecular damage, yet this conventional view leaves much unexplained. Over time, cells and tissues appear to lose the regulatory flexibility that allows them to adapt, repair, and reconfigure their functional states. Genomic instability, metabolic imbalance, mitochondrial dysfunction, and proteostatic decline converge on aging, but their effects focus on chromatin organization, transcriptional coordination, and signaling networks that maintain cellular identity. In this review, we propose that aging can be usefully viewed as a progressive restriction of epigenetic and regulatory plasticity, rather than as the simple accumulation of lesions. Pathways such as Wnt signaling, TET-dependent DNA demethylation, and metabolic sensors including AMPK, mTOR, and sirtuins create an interconnected landscape that links environmental and metabolic conditions with long-term cellular behavior. As this landscape becomes increasingly rigid and constrained, cells retain viability but lose their capacity for dynamic responses, stabilizing in low-plasticity states that include cellular senescence. Framing aging as a shift from adaptive plasticity toward regulatory rigidity offers a possible integrative lens on classical hallmarks and epigenetic aging signatures, without replacing existing models. Rather than targeting individual hallmarks in isolation, future approaches may need to complement hallmark-focused strategies by restoring dynamic balance within epigenetic and signaling networks that preserve tissue-level homeostasis and regenerative potential, thereby suggesting specific, testable predictions for interventions acting on metabolic–epigenetic axes.

## Introduction

Aging manifests as declining organismal function-yet this observation reveals little about underlying mechanisms. Although numerous molecular mechanisms have been implicated (from genomic instability to metabolic dysregulation), the question of what truly drives aging remains open (López-Otín et al., 2023). The difficulty does not stem from a lack of mechanisms, but in their interdependence: cellular systems do not degrade in isolation but as components of a densely interconnected regulatory network.

Classical views emphasize the progressive accumulation of molecular damage. However, systems responsible for DNA repair, proteome maintenance, and regulatory precision are maintained only within functional limits rather than optimized for unlimited stability. Over time, even minor deficiencies in these systems begin to amplify one another, gradually disrupting cellular homeostasis. Viewed differently, aging cannot be reduced to a simple accumulation of molecular damage. It reflects a slow erosion of regulatory equilibrium.

**FIGURE 1:**
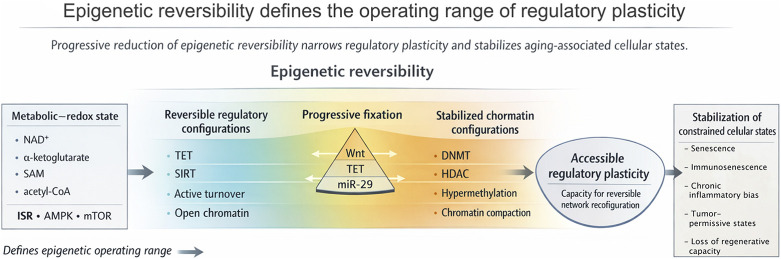
Conceptual model of aging as contraction of regulatory state space. Persistent molecular and metabolic perturbations progressively constrain the range of accessible regulatory configurations within cellular networks, shifting the system from flexible, compensatory remodeling toward increasingly restricted, low-plasticity states. These constrained states are typically associated with senescence, chronic inflammatory signaling and reduced regenerative capacity. Interventions targeting metabolic, epigenetic, or senescence-associated processes may transiently reopen a window of reversibility. Arrows indicate conceptual relationships inferred from existing literature rather than universally established causal pathways.

In this review, we propose that aging is best understood as a progressive restriction of epigenetic and regulatory plasticity. Epigenetic regulation, located at the interface of the genome, environment, and cellular identity, appears particularly central, because it integrates diverse molecular perturbations into long-term changes in cell fate. We synthesize evidence across hallmarks of aging, epigenetic drift, metabolic regulation, cellular senescence, and immunoaging to argue that functional decline correlates more closely with loss of adaptive regulation than with the burden of damage itself (see Figure 1).

In this narrative and conceptual Review, we do not aim to provide an exhaustive catalogue of all mechanisms implicated in aging. Instead, we selectively synthesize evidence across classical hallmarks of aging, epigenetic drift and clocks, chromatin architecture, metabolic regulation, cellular senescence and immunoaging, together with illustrative axes such as Wnt signaling, TET-dependent DNA demethylation and microRNA networks. Our goal is to examine how these processes converge on epigenetic and regulatory control systems, and to develop a testable framework in which progressive restriction of regulatory plasticity provides an integrative perspective on aging rather than a replacement for existing models. Conceptually, we organize the discussion along a sequence in which molecular damage and stress inputs are first integrated by metabolic and epigenetic systems, progressively narrowing the accessible regulatory state space, leading to stabilized low-plasticity states such as senescence and immune remodeling, and finally revealing potential intervention points that can transiently reopen plasticity without destabilizing cellular identity.

## Revisiting the hallmarks of aging: from damage to regulatory constraint

Aging at the tissue level emerges from the gradual limitation of cellular pathways that sustain genomic stability, proteome integrity, and coordinated signaling. Although classical hallmarks of aging are often described separately, in practice they form a tightly interconnected network of constraints that progressively restrict cellular flexibility. Table 1 summarizes how each classical hallmark can be interpreted not only as damage but as an emergent constraint on regulatory plasticity.

**TABLE 1:** Aging as a progressive constraint on regulatory plasticity: conceptual mechanisms prioritizing stability over reversibility.

Hallmark	Damage	Constraint on regulatory plasticity	Unresolved issues
Genomic instability	Accumulation of somatic mutations impairs cellular function	Accumulation of mutations alone may be insufficient to account for aging. Chromatin maintenance and replication control improve repair fidelity but also allow damaged sequences to persist, and over time this can narrow the range of regulatory states that a cell can access	Relative contribution of mutation burden vs. chromatin/repair reprogramming to functional decline in human tissues
Telomere attrition	Telomere shortening limits proliferation and promotes senescence	Telomere dysfunction generates persistent damage signaling. The limitation may lie less in irreparability than in the way cells choose between repair, apoptosis and arrest, often favoring prolonged cell-cycle arrest rather than a flexible resolution of stress	When telomere-driven arrest is protective vs. maladaptive; tissue- and cell-type specificity
Epigenetic alterations	Global changes in chromatin structure, DNA and histone modifications, splicing, and translational control	Developmental and stochastic epigenetic drift reinforce existing lineage programs. Over time, increased reliance on DNA methylation together with reduced chromatin remodeling can make it harder to reconfigure transcriptional networks, thereby limiting dedifferentiation and regeneration	Extent to which age-related epigenetic drift is cause, amplifier or marker of loss of plasticity
Loss of proteostasis	Accumulation of misfolded and damaged proteins disrupts cellular systems	Core quality-control pathways (autophagy, mitophagy, proteasomal degradation) are rarely absent; their activity is more often insufficient. As a result, protein turnover becomes too slow to support effective proteome renewal or a broader molecular reset after stress	Whether slower turnover reflects energy conservation, impaired renewal, or both
Deregulated nutrient sensing	Chronic overactivation of growth and anabolic pathways suppresses repair and maintenance	Sustained anabolic signaling locks cells into metabolic and transcriptional states that are difficult to reverse. Loss of oscillatory inputs and environmental variability further pushes cells towards energetically stable but less adaptable regulatory states	How much plasticity can be restored by reintroducing metabolic oscillations in aged tissues
Mitochondrial dysfunction	Excessive ROS production and respiratory defects damage cellular components	Beyond their bioenergetic role, mitochondria influence epigenetic regulators, differentiation decisions and stress-response pathways. When mitophagy is impaired and defense programs remain chronically active, cellular states tend to become stabilised and transitions between them occur less readily	Driver vs. compensatory remodeling; which mitochondrial changes are reversible *in vivo*
Cellular senescence	Accumulation of irreversibly arrested cells due to unresolved damage	Senescent states are reinforced by persistent signaling, including SASP, and by reduced immune clearance. In this setting, cells become effectively locked in a non-proliferative state even though they remain metabolically active	Net balance of beneficial vs. deleterious senescent cell functions in different contexts
Stem cell exhaustion	Reduced capacity to replenish differentiated cells and maintain tissue homeostasis	Some self-renewal potential often remains, whereas differentiation capacity and lineage choice become increasingly restricted. Evidence that aged stem cells can recover function under altered niche or systemic conditions suggests that exhaustion mainly reflects constrained state transitions rather than a complete loss of intrinsic potential	Degree to which exhaustion reflects intrinsic damage vs. extrinsic niche and systemic constraints
Inflammaging and altered intercellular communication	Chronic low-grade inflammation disrupts systemic coordination	Persistent cytokine and danger signaling gradually reinforces maladaptive activity patterns across tissues. Positive feedback loops between immune cells, senescent cells and stromal compartments make it increasingly difficult for regulatory networks to return to baseline or initiate regenerative responses	How far systemic inflammatory set points can be reset without impairing host defense

### Somatic DNA damage and genomic integrity

Somatic mutations accumulate throughout life, yet their precise contribution to aging remains uncertain (Blokzijl et al., 2016; Manders et al., 2021; Milholland et al., 2017; Moore et al., 2021; Ren et al., 2024). Severe impairment of DNA maintenance systems, as observed in progeroid syndromes such as Werner syndrome, clearly produces rapid cellular dysfunction and premature tissue decline (Zhang et al., 2015; Oshima et al., 2017). However, the relatively steady accumulation of mutations during normal aging does not produce equally dramatic effects (Robinson et al., 2021).

This divergence points to the fact that the core problem may lie less in the presence of genetic lesions themselves and more in the declining capacity of cellular systems to maintain functional proteomes and stable gene expression in their presence - a decline likely rooted in dysregulated epigenetic control, altered transcription, impaired autophagy, and metabolic imbalance.

### Telomere dynamics and replicative constraint

In cellular aging, telomeres undergo gradual shortening with successive divisions and accumulate damage that is particularly difficult to fix in the context of chromatin. The shelterin complex protects chromosome ends from being recognized as double-strand breaks, yet at the same time restricts access of classical repair pathways, allowing persistent lesions to trigger chronic activation of the DNA damage response (Ruis and Boulton, 2021).

Thus, telomeres become a source of maintained stress signals that promote stable cell cycle arrest and senescence (de Lange, 2005; Ruis and Boulton, 2021). This replicative limitation serves a protective, anti-oncogenic function, as only stem and selected progenitor cells retain long-term proliferative capacity. However, reduced telomerase activity and progressive telomere attrition can gradually impair tissue renewal and disturb homeostasis.

Crucially, telomere length alone does not reliably predict lifespan, indicating that telomere dynamics represent one regulatory constraint among many shaping cellular and tissue aging (Codd et al., 2021).

### Mitochondria as regulators of cellular stress and adaptation

Free radicals serve dual roles - as signaling molecules and destructive agents. The telomeric sequence (TTAGGG), rich in guanine and highly susceptible to oxidation, illustrates this dual role. Telomerase does not repair such damage, and the shelterin complex offers little protection against reactive oxygen species (ROS) (Passos et al., 2007). Interestingly, under oxidative stress the protein subunit of telomerase, TERT, can assume a protective role within mitochondria. By associating with mitochondrial DNA, it enhances respiratory chain activity and limits damage (Haendeler et al., 2009; Ahmed et al., 2008).

Contrary to the classical view of ROS as primary drivers of aging, numerous studies have shown little direct correlation between ROS levels and organismal lifespan. Although oxidative stress undoubtedly impairs cellular function, it appears to represent more a symptom and signaling mediator than a fundamental cause of aging (Trifunovic et al., 2005; Dai et al., 2010; Budinger and Chandel, 2025). Among these, the maintenance of efficient autophagy (particularly mitophagy) is of central importance, as it preserves mitochondrial quality and supports metabolic stability (Ho et al., 2017).

### Proteostasis and regulatory fidelity

Age-related transcriptional noise disrupts proteome maintenance, leading to progressive failure of multiple cellular systems. Insufficient clearance of aberrant protein species promotes the formation of cytotoxic aggregates, widely recognized as a feature of numerous age-associated disorders, including neurodegenerative diseases (Johnson et al., 2025; Pickrell and Youle, 2015; van Ham et al., 2010).

These disturbances often arise from impairments in ubiquitination and protein clearance pathways, which also interfere with mitophagy and contribute to energy imbalance and excessive ROS production. Mitophagy plays a crucial role in preserving mitochondrial integrity and maintaining cell metabolic capacity. It is also essential for sustaining the self-renewal potential of stem cells (Ho et al., 2017; Vannini et al., 2016).

Proteostasis is tightly regulated by molecular chaperones that aid correct protein folding and coordinate the refolding or degradation of damaged proteins through the ubiquitin-proteasome system and chaperone-mediated autophagy. Under proteotoxic stress conditions, including oxidative damage, expression of chaperone genes increases markedly to maintain protein quality control (Hartl et al., 2011).

In long-lived postmitotic cells such as neurons and cardiomyocytes, the progressive buildup of damaged macromolecules (including defective mitochondria, cytosolic aggregates, and lipofuscin) reflects the limited efficiency of cellular degradation systems (Terman et al., 2010). Lipofuscin, a chemically stable end product of lipid and protein oxidation, accumulates within lysosomes and interferes with autophagic processes (Brunk and Terman, 2002). Age-related alterations in nuclear lamina components, including low-level production of aberrant lamin A isoforms, further destabilize chromatin organization and genome integrity. (Krishnan et al., 2011; Ragnauth et al., 2010).

### Metabolic oscillation and allostatic balance

**FIGURE 2:**
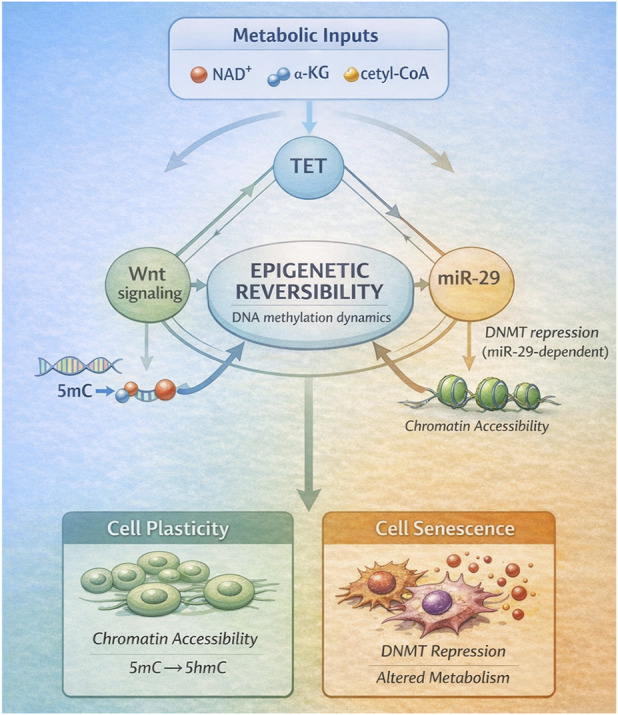
Epigenetic regulation of cellular plasticity through the Wnt–TET axis. The canonical Wnt/β-catenin signaling pathway can modulate epigenetic plasticity by influencing chromatin accessibility and DNA methylation dynamics, and in some contexts promotes transcriptional programs associated with stemness and tissue regeneration. Wnt signaling interacts with epigenetic regulators such as TET dioxygenases, which oxidize 5-methylcytosine (5 mC) to 5-hydroxymethylcytosine (5hmC) and facilitate DNA demethylation and transcriptional activation. Through this interplay, Wnt signaling integrates environmental and metabolic signals to maintain cellular plasticity and regenerative capacity. Solid arrows denote interactions supported by multiple experimental studies, whereas the overall scheme is intended as an interpretive model highlighting plausible points of crosstalk.

The metabolic state of the cell influences virtually all other processes, including DNA repair (Moretton and Loizou, 2020; Lagunas-Rangel, 2019). The relationship between metabolic state, epigenetic reversibility and regulatory plasticity is summarized in Figure 2.

There is no universal equilibrium; under natural conditions organisms function in a state of dynamic allostasis, constantly adjusting physiological parameters in ways that also shape the epigenetic operating range of cells (Figure 2). When metabolic oscillations associated with physical activity or periodic nutrient scarcity are lost, cells drift toward a more static energetic state and gradually forfeit the adaptive benefits of metabolic flexibility. Caloric restriction remains the most robust intervention known to extend lifespan or maintain metabolic health across multiple species (Colman et al., 2009).

Sirtuins, particularly SIRT1, function as key metabolic and epigenetic regulators whose activity depends on intracellular NAD^+^ availability. Their importance for longevity appears to proceed from their ability to coordinate metabolic state with transcriptional control, autophagy, and mitochondrial function. SIRT1 promotes autophagy through deacetylation of FOXO transcription factors, enhances mitochondrial biogenesis via PGC-1α, and interacts functionally with AMPK and other nutrient-sensing pathways (Zhang X. et al., 2021; Lee et al., 2008; Gupta et al., 2025; Fernandez-Marcos and Auwerx, 2011; Cantó et al., 2009).

Because of its relatively low affinity for NAD^+^, SIRT1 acts as a sensitive sensor of cellular energy balance. Age-associated declines in NAD^+^ levels, together with competition from other NAD^+^-consuming enzymes such as PARP1, disrupt this regulatory axis and may shift DNA repair and chromatin regulation toward less stable states (Chung and Joe, 2014; Chen Y. et al., 2019).

Beyond nuclear regulation, many crucial cellular decisions are made at the cytosolic level, including the balance between survival, apoptosis, and adaptive stress responses. The integrated stress response (ISR), triggered by nutrient deprivation, mitochondrial dysfunction, viral signals, or proteotoxic stress, suppresses global protein synthesis while enabling selective translation of stress-adaptive factors such as ATF4. This mechanism links metabolic state directly to transcriptional reprogramming and cellular adaptation (Kim et al., 2013; Jang et al., 2021; Mick et al., 2020).

Rather than acting as isolated drivers of aging, these hallmarks collectively reflect the gradual loss of the regulatory flexibility that maintains cellular identity and adaptive capacity.

## Metabolism as an architect of the epigenetic landscape

Although these classical hallmarks describe diverse forms of cellular damage and dysfunction, many of them converge on a common consequence: progressive destabilization of regulatory and epigenetic control systems that maintain cellular identity.

Cellular metabolism exerts a direct and pervasive influence on the epigenetic landscape. NAD^+^ and ATP integrate the energetic and redox state of the cell with chromatin regulation, creating a functional bridge between metabolic activity and gene expression. During aging, transient metabolic stress as well as chronic mitochondrial dysfunction progressively disrupt this balance, not merely through substrate depletion but through alterations in the availability and ratios of key metabolic cofactors required for epigenetic enzymes (Ummarino et al., 2021; Lee et al., 2008; Srivastava, 2016).

The activity of TET dioxygenases, for example, depends on the availability of α-ketoglutarate and on the balance between α-ketoglutarate and metabolites such as succinate and fumarate. DNA and histone methyltransferases respond to the S-adenosylmethionine to S-adenosylhomocysteine ratio linked to one-carbon metabolism, whereas histone acetyltransferases rely on nuclear acetyl-CoA pools. In this way, fluctuations in mitochondrial and cytosolic metabolism are continuously translated into changes in chromatin organization and transcriptional activity (Etchegaray and Mostoslavsky, 2016; Yang et al., 2023).

Under conditions of persistent metabolic imbalance, the epigenetic landscape gradually shifts toward an adaptive, energy-conserving configuration. Initially protective, but this shift may, over time, limit transcriptional flexibility.

Nutrient-sensing pathways also influence RNA processing. Activation of mTORC1 and downstream kinases such as S6K1 disrupts splicing regulators including SRPK2, thereby linking metabolic state with the production of functional mRNA isoforms (Lee et al., 2017). Conversely, AMPK-mediated phosphorylation of TET2 protects it from degradation and supports epigenetic stability, illustrating how metabolic signaling directly modulates chromatin-modifying enzymes (Wu et al., 2018; Zhang et al., 2019).

Competition for NAD^+^ between sirtuins and PARP enzymes provides another layer of regulation. While PARP1 is indispensable for DNA repair, its excessive activation can deplete NAD^+^ pools and alter chromatin organization and inflammatory signaling. At the same time, interactions among PARP1, DNMT1, and CTCF demonstrate how metabolic state modulate regional DNA methylation patterns and genome architecture (Ummarino et al., 2021; Morales et al., 2014; Hassa, 2009; Zampieri et al., 2012; Ong et al., 2013).

Sirtuins further contribute to the stabilization of chromatin structure and genome topology. Proteins such as SIRT6 and SIRT7 help maintain heterochromatin integrity and repress transposable elements, while mitochondrial sirtuins participate in nuclear–mitochondrial communication and epigenetic regulation (Bi et al., 2020; Van Meter et al., 2014; Diao et al., 2021). Even the mitochondrial genome itself exhibits epigenetic modulation, and metabolic interventions such as physical activity can alter mtDNA methylation and respiratory function (Ruple et al., 2021).

**FIGURE 3:**
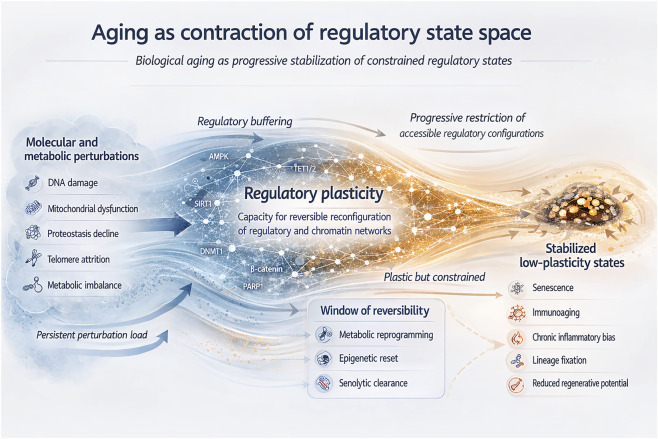
Metabolic and regulatory control of epigenetic reversibility. Metabolic cofactors such as NAD^+^, α-ketoglutarate (α-KG) and acetyl-CoA influence epigenetic remodeling by modulating the activity of chromatin-modifying enzymes. TET dioxygenases integrate these metabolic signals with regulatory pathways including Wnt signaling and miR-29–mediated repression of DNA methyltransferases (DNMTs), shaping DNA methylation dynamics and chromatin accessibility. Together, these interactions determine whether cells maintain regenerative plasticity or shift toward senescence-associated phenotypes. Solid arrows denote interactions supported by multiple experimental studies, whereas the overall scheme is intended as an interpretive model highlighting plausible points of crosstalk. The figure does not attempt to capture all regulators of epigenetic reversibility, but highlights one set of experimentally supported interactions that exemplify how metabolic and signaling inputs can converge on DNA methylation dynamics.

Collectively, these observations indicate that metabolic regulation does not merely support cellular activity but continuously reshapes the epigenetic framework that determines cellular identity and flexibility. If metabolism shapes the epigenetic landscape in real time, then aging reflects not only damage accumulation but the progressive fixation of these metabolically imprinted states. The regulatory circuitry linking metabolic inputs, chromatin dynamics, and cell fate decisions is summarized in Figure 3.

In this Review, the term “regulatory plasticity” denotes the ability of cellular regulatory networks–encompassing chromatin states, transcriptional programs, signaling pathways and cell-identity circuits–to move between distinct yet viable configurations in response to perturbations and then return to an adaptive attractor state. This usage is related to, but distinct from, concepts such as epigenetic drift (stochastic change in marks), resilience (organism-level recovery), stem cell exhaustion (loss of progenitor pools), stable senescent states or loss of cellular identity. Reduced regulatory plasticity in our sense refers to a narrowing of the accessible regulatory state space, in which transitions become less frequent, less reversible or more constrained even when baseline viability is preserved. Importantly, some restriction of plasticity can be adaptive–for example, by stabilizing lineage identity or limiting malignant transformation–so regulatory rigidity should be viewed as a context-dependent feature rather than a uniformly detrimental one.

From this perspective, classical features of aging can be interpreted not only as damage, but as cumulative faults that gradually limit the adaptive scope of regulatory networks (Tabrez et al., 2017; Li and Tollefsbol, 2016).

This shift in emphasis raises a practical question: should biological aging be assessed primarily by the burden of damage, or by the declining capacity of regulatory systems to reconfigure and recover? Operationally, this “capacity to reconfigure and recover” could be quantified using perturbation–response assays rather than static measurements alone. Relevant readouts may include: (i) recovery time of transcriptional profiles after a standardized stress (for example, cytokine or oxidative pulses) measured by bulk or single-cell RNA-seq; (ii) changes in chromatin accessibility or histone-mark landscapes (ATAC-seq, ChIP-seq) in response to transient metabolic or inflammatory challenges; (iii) reversibility of single-cell trajectories in differentiation or reprogramming models; and (iv) restoration of mitochondrial function and metabolic fluxes after acute insults. Such dynamic measures could be combined with epigenetic or chromatin-accessibility clocks to ask whether tissues of similar “molecular age” differ in their regulatory responsiveness. Together, these measurements characterize the dynamic capacity of regulatory networks as the speed and completeness with which they return to adaptive configurations after transient perturbation.

## Epigenetic drift and structural destabilization

Beyond fixed genomic damage and the accumulation of macromolecular aggregates, cellular aging increasingly appears to converge on epigenetic regulation. While epigenetic mechanisms are unlikely to function independently; rather may represent the level at which diverse molecular perturbations are integrated and translated into long-term changes in cellular identity.

### DNA methylation dynamics and epigenetic clocks

A classical example of an epigenetic clock is the widely used Horvath clock, based on 353 CpG sites, which highlights that age-associated changes preferentially affect regions involved in cellular homeostasis and proliferation. With aging, selected CpG sites within promoters and regulatory regions of growth- and cell cycle–associated genes tend to gain methylation, whereas regions linked to embryonic development, cell identity, and plasticity often exhibit relative hypomethylation.

The rate of epigenetic change correlates with proliferative dynamics, being highest during early development and progressively slowing in adulthood. Notably, no significant difference in methylation age has been observed between embryonic stem cells and induced pluripotent stem cells, supporting the concept that the epigenetic clock can be reset (Horvath, 2013).

It is hence appropriate to speak of both hypermethylation and hypomethylation during aging; however, the Horvath clock does not measure gene expression directly. Many CpG sites included in the clock are not located within promoters and do not show a simple linear relationship with transcriptional activity.

As aging progresses, the activity of the Polycomb Repressive Complex 2 (PRC2) declines. PRC2 mediates transcriptional repression through trimethylation of histone H3 at lysine 27 (H3K27me3), creating a layer of facultative heterochromatin capable of dynamic regulatory responses. When this regulatory layer weakens, it is often replaced by DNA methylation, which is considerably less flexible. Loss of H3K27me3 can induce expression of p16 (CDKN2A) independently of overt DNA damage, thereby promoting replicative senescence (Ito et al., 2018).

Additionally, reduced PRC2 activity may reflect broader functional impairment of its components. For example, EZH2 has been implicated in processes extending beyond histone methylation, including DNA replication (Ito et al., 2018). Epigenetic aging thus reflects a shift toward less flexible chromatin configurations…

### Chromatin architecture and regulatory stability

The topology of the entire genome is as important as the chemical modification of DNA. Chromatin changes defy simple categorization. While H3K27me3 marks increase globally, although heterochromatin is lost in strategic locations. Structural reorganization disrupts mostly intergenic regions, including enhancers (Zhang C. et al., 2021). Chromatin reorganization can, for example, activate placental-specific genes, contributing to cellular aging (Liu et al., 2022).

An additional layer of regulation is reflected in clocks based on chromatin accessibility (Morandini et al., 2024). Open chromatin regions (OCRs) change their accessibility during aging, indicating that epigenetic drift is not limited to methylation patterns but extends to the broader regulatory architecture of the genome (Thakur et al., 2023).

An important feature of chromatin organization is the existence of Topologically Associating Domains (TADs)-large chromatin units in which DNA sequences interact more frequently within the domain than outside it. TADs define the boundaries within which regulatory elements can act. Their formation and maintenance rely primarily on loop extrusion mediated by cohesin, with CTCF controlling loop positioning and stabilization (Dehingia et al., 2025). Disruption of these structures alters regulatory insulation and disrupts loci such as INK4/ARF, which is critical for proliferative control and senescence (Hirosue et al., 2012).

Lamina-associated domains (LADs) further participate in the regulation of gene expression. Loss of Lamin B1 produces derepression of LAD regions. Upon relocation away from the nuclear periphery, these regions become accessible to p300, enabling the formation of *de novo* enhancers that contribute to inflammaging (Sen et al., 2019; Lasko et al., 2017).

Replicative and oncogene-induced senescence is characterized by extensive chromatin remodeling in LAD regions, including enrichment of H3K4me3 and the emergence of H3K27me3-depleted “canyons.” These regions are rich in genes and enhancers, and their activation links senescence-associated chromatin reorganization with premalignant epigenetic states (Shah et al., 2013).

A fraction of the autophagy protein LC3 localizes to the nucleus, where it associates with Lamin B1 and lamina-associated domains. Under persistent oncogenic signaling, LC3 can facilitate Lamin B1 degradation via an autophagy-dependent pathway, reinforcing LAD disorganization and stabilizing oncogene-induced senescence (Dou et al., 2015).

### Transcriptional noise and regulatory drift

Lifespan changes arise from both programmed regulation and stochastic error. Transcriptional noise disrupts cellular function and drives genetic drift-the progressive uncoupling of individual cells within a tissue.

Derepression of transposons (Yushkova and Moskalev, 2023; Sturm et al., 2023), expression of proteins inappropriate for tissue specificity (Yanai et al., 2006), altered splicing patterns, and replication- or repair-associated perturbations all create the potential for regulatory instability. These processes may be further amplified by chronic anabolic signaling, sexual maturation, or persistent immune activation.

In chondrocytes, stimulation with proinflammatory cytokines significantly decreases DNMT1 expression (Hashimoto et al., 2009); similar effects have been observed in the context of vascular insulin resistance (Balakrishnan et al., 2018). However, in cancer cells, IL-6 increases DNMT1 levels, promoting hypermethylation of tumor suppressor gene promoters (Foran et al., 2010). More generally, DNMT1 expression often declines with age, contributing to loss of maintenance methylation during replication, while DNMT3A and DNMT3B expression may increase and promote local *de novo* methylation of CpG islands (Uysal and Ozturk, 2020; Xiao et al., 2008; Ciccarone et al., 2016; Li Q. et al., 2020).

### Alternative splicing and transcriptome remodeling

Alternative splicing increases with age (Rodríguez et al., 2016). Exon skipping and intron retention are among the most common age-associated changes (Dong et al., 2018). These alterations support the view that splicing dysregulation constitutes a significant component of transcriptional noise in aging cells.

The tumor suppressor p53 provides a striking example. DNA damage promotes the generation of the p53β isoform, which is characteristic of cellular senescence and exhibits reduced proapoptotic activity compared with full-length p53 (Chen et al., 2021). The balance between p53β and Δ133p53 isoforms influences proliferative capacity and senescence progression (Fujita et al., 2009; Horikawa et al., 2017).

Splicing alterations also affect regulators of p53 stability. Reduced levels of the splicing factor SRSF7 in senescent cells promote the generation of a shortened MDM2 isoform incapable of properly regulating p53 (Hong J. et al., 2023).

SIRT1 itself is subject to alternative splicing. Only the full-length SIRT1-v1 isoform enhances aerobic respiration, and its levels decline during cardiac aging (Zhang X. et al., 2021). Stress-dependent shifts in SIRT1 isoform expression have been associated with functional decline in neural tissues (Shlomi et al., 2022; Sarne et al., 2018).

DNA methylation influences exon inclusion, whereas TET enzymes can facilitate exon recognition through interactions with CTCF and modulation of RNA polymerase II progression (Marina et al., 2016; Marina and Oberdoerffer, 2016).

With aging, a general increase in RNA polymerase II elongation rate has been observed. This acceleration, linked to global chromatin remodeling and histone loss, alters transcriptional fidelity. Experimental slowing of transcription through increased histone abundance extends lifespan in model organisms (Debès et al., 2023).

Chronic DNA damage signaling, potentially arising from telomere dysfunction, drives histone depletion and further destabilizes transcriptional regulation (O’Sullivan et al., 2010). The scale of changes resulting from splicing alterations is further amplified by the generation of circular RNA (circRNA). Their levels increase with age in neuronal tissues of many animal species, although their impact on cellular function may be both beneficial and detrimental (Weigelt et al., 2020).

Until recently, the study of transcriptional noise at the single-cell level was limited by the lack of appropriate sequencing technologies. Analyses of gene expression in whole tissues provided only a coarse assessment of epigenetic drift. A model of gene expression predictability based on regulatory network correlations (Leote et al., 2024) enabled evaluation of whether expression patterns within a given tissue became more chaotic or more ordered with age. Predictability was defined by the degree to which expression of a gene remained correlated with that of its regulators. Notably, in some cases predictability increased with age, suggesting a reduction in stochastic noise. Older individuals displayed more synchronized regulation of genes involved in coagulation and anti-inflammatory processes, which may represent an adaptive feature. At the same time, these observations illustrate a progressive loss of flexibility at key regulatory nodes. Coordination within mitochondrial metabolic networks weakened, and genes such as LAMTOR5, involved in mTORC1 signaling, lost correlation with many of their regulators and neighboring genes (Leote et al., 2024). Although these findings were derived from tissue-level analyses, they highlight a shift from flexible regulatory states toward more constrained transcriptional programs.

Studies using single-cell transcriptomics suggest that transcriptional instability is not universally increased with age (Warren et al., 2007); however, such conclusions are often based on limited datasets and do not account for alternative splicing, transposon derepression, system uncoupling, or the growing production of defective transcripts subsequently removed by nonsense-mediated decay (NMD). In autoimmune diseases, abnormal DNA and histone methylation patterns affect genes involved in interferon and TNF signaling, cell survival, metabolism, and antibody production (Zheng and Sawalha, 2022). In pancreatic islet endocrine cells from older donors, increased transcriptional noise and epigenetic drift lead to the expression of genes and hormones inappropriate for cell identity, potentially contributing to declining organ function (Enge et al., 2017).

Splicing errors frequently result in intron retention, introducing premature stop codons that activate the NMD pathway. Because NMD efficiency itself declines with age, abnormal transcripts may accumulate and contribute to proteostatic imbalance. Approximately 75% of mammalian genes with multiple exons exhibit intron retention as part of physiological regulation, yet its frequency increases with age (Baralle and Romano, 2023).

A well-characterized example is the endoglin (CD105) gene encoding the TGF-β co-receptor. Alternative splicing generates two isoforms: a long, proangiogenic form predominant in young endothelial cells and a shorter isoform with opposing effects. Age-associated intron retention favors production of the shorter form, potentially contributing to endothelial dysfunction and vascular pathologies such as hypertension (Baralle and Romano, 2023).

### Systemic and developmental influences on the epigenome

MicroRNA-mediated regulatory networks provide an additional layer of epigenetic control. miR-29 acts as a key regulator of epigenetic homeostasis, maintaining appropriate levels of DNMT and TET enzymes and stabilizing the differentiated state. Its expression increases with age and under oxidative or inflammatory stress, potentially reinforcing epigenetic rigidity (Morita et al., 2013; Tu et al., 2015). Similarly, ΔNp63 maintains the youthful phenotype of keratinocytes by repressing specific microRNAs. Decline of ΔNp63 during replicative stress or DNA damage releases this repression, promoting SIRT1 inhibition and senescence-associated regulatory loops (Rivetti di Val Cervo et al., 2012).

Age-associated epigenetic changes also reflect systemic and ontogenetic influences. Signals from the germline and endocrine system may modulate somatic aging trajectories, including age-dependent decline of mitochondrial stress responses and thymic involution (Zhou et al., 2024; Polesso et al., 2023). Additionally, stress-dependent alterations in small noncoding RNAs within germ cells may influence epigenetic programming of the next-generation (Duffy et al., 2021).

As regulatory and epigenetic flexibility declines, cells may transition into stable functional states characterized by persistent signaling imbalance and limited reversibility, ultimately manifesting as cellular senescence.

## Cellular senescence as a stabilized state of reduced plasticity

Senescent cells arrest irreversibly but remain metabolically active. They exhibit persistent DNA damage, mitochondrial dysfunction, and shortened telomeres, and express high levels of cell cycle inhibitors while secreting a range of pro-inflammatory factors and proteases collectively known as the senescence-associated secretory phenotype (SASP). Crucially, clearance of senescent cells can stimulate stem cell proliferation and delay the onset of age-related phenotypes (Roger et al., 2021).

In the context of the loss of cellular plasticity we have been considering, senescent cells take on a somewhat paradoxical character - representing, in a sense, the culmination of this process pushed to its extreme. Replicative senescence was first described by Hayflick through serial passaging of cells, with telomere shortening identified as a primary limiting factor (Bodnar et al., 1998; Hayflick and Moorhead, 1961). However, physiological cellular aging appears only partially dependent on telomere length and is more closely linked to epigenetic regulation, remaining to some extent reversible (Shimamoto et al., 2014; Su et al., 2023).

In stem cell populations, telomere attrition seems to play a relatively minor role compared with disturbances in asymmetric division, loss of niche interactions, lineage bias, and declining self-renewal capacity. This loss is often concealed: stem cells may appear functional, yet subtle epigenetic changes reduce their regulatory “plasticity.” Aging in these cells is closely associated with the accumulation of DNA damage. Impaired DNA damage response (DDR) and reduced apoptotic sensitivity may confer a selective advantage to aged stem cells, allowing their persistence within tissues. Many stem cells reside in a quiescent G0 state in which non-homologous end joining predominates. However, this mechanism alone does not fully explain the accumulation of DNA damage markers such as γ-H2AX in aging hematopoietic stem cells. Quiescent cells may accumulate damage while awaiting cell cycle entry, at which point homologous recombination can occur; when this process becomes dysregulated, it drives functional stem cell decline (Iordache et al., 2024; Gutierrez-Martinez et al., 2018; Beerman et al., 2014; Insinga et al., 2013).

SASP itself is not solely detrimental. During tissue repair, it initially supports wound closure through an immunosuppressive, profibrotic secretome characterized by TGF-β and PDGF-AA expression. As healing progresses, the secretory profile shifts toward a pro-inflammatory and fibrolytic phenotype dominated by IL-1β, IL-6, and IL-8, facilitating immune clearance of senescent cells and extracellular matrix remodeling. The NOTCH pathway plays a strategic role in orchestrating this transition. High NOTCH1 activity suppresses pro-inflammatory SASP, whereas its decline promotes cytokine expression, lymphocyte recruitment, and immune surveillance of senescent cells (Demaria et al., 2014; Hoare et al., 2016; Ito et al., 2017).

Senescent cells also communicate with neighboring cells through extracellular vesicles containing proteins and RNA, contributing to the so-called bystander effect, in which surrounding cells acquire senescence-like features (Olivieri et al., 2015). These processes are further modulated by systemic factors. Experimental paradigms such as parabiosis or transfusion of blood from younger organisms into older ones demonstrate that circulating signals can partially restore regenerative capacity (Irina Conboy and Rando, 2012).

The canonical Wnt/β-catenin pathway, illustrates the context-dependent nature of senescence. For instance, WNT9A signaling in renal tubular epithelial cells induces senescence and fibrosis (Congwei et al., 2018), and similar effects are observed in the lung under sustained pathway activation (Lehmann et al., 2020). Conversely, under physiological conditions, Wnt signaling may prevent senescence in mesenchymal stem cells and support regenerative responses (Lehmann et al., 2022).

In skeletal muscle regeneration, Wnt/β-catenin activity is required for progenitor differentiation but is tightly regulated through interactions with the NOTCH pathway. The NOTCH intracellular domain can form nuclear complexes with β-catenin and repress its transcriptional activity, thereby balancing proliferation and differentiation (Acar et al., 2021). Pro-inflammatory signals such as TNFα disrupt this regulatory axis by inhibiting NOTCH activation, impairing regenerative capacity (Brack et al., 2009; Brack et al., 2008; Acharyya et al., 2010). MicroRNAs further modulate this interplay; for example, miR-29 levels increase in response to Wnt3A and may promote muscle cell aging (Hu et al., 2014). Notch signaling also maintains stem cell quiescence and supports niche integrity, acting in concert with Wnt when not dominant (Koch et al., 2013; Bjornson et al., 2012).

Systemic modulators add further levels to regulation. The protein Klotho, in its secreted form, binds Wnt ligands such as Wnt3A and functions as an endogenous inhibitor of canonical signaling. Loss of Klotho results in premature aging phenotypes, whereas its overexpression extends lifespan in mice (Kuro-o et al., 1997; Liu et al., 2007).

Components of SASP can directly modulate stem cell fate. The secreted form of PTK7, for example, activates non-canonical Wnt/Ca^2+^ signaling in intestinal stem cells and disrupts asymmetric division patterns (Yun et al., 2023). Chronic inflammation may further contribute to aging independently of senescent cell burden. Inhibition of the NLRP3 inflammasome extends lifespan in experimental models, reflecting not only suppression of SASP but also broader attenuation of inflammatory signaling and oxidative stress (Marín-Aguilar et al., 2020; Krishnan et al., 2019).

## Immunoaging and inflammatory regulation

The inability to efficiently eliminate cells that should be removed from tissues appears to be one of the central problems of aging. Apoptotic potential declines for multiple reasons, including increased expression of anti-apoptotic proteins of the Bcl-2 family or inhibitors such as surviving (Sasaki et al., 2001; Zhu et al., 2016; Ma et al., 2016; Al-Khalaf and Aboussekhra, 2013). Even when intrinsic cellular control mechanisms fail, tissue integrity should in principle be protected by cytotoxic lymphocytes, raising doubts about steady erosion in immune surveillance.

With age, the number of defective or dysfunctional cells increases in tissues, yet their removal may also intensify, partly in response to derepression of endogenous viral elements (Hasegawa et al., 2023). In supercentenarians, the immune system often shifts toward a more cytotoxic profile, with fewer B lymphocytes and an expanded pool of cytotoxic cells, including atypical CD4^+^ populations exhibiting effector functions (Hashimoto et al., 2019). At the same time, elevated levels of regulatory T cells suggest that immune flexibility, rather than simple activation, is a hallmark of healthy aging (Zhou et al., 2022).

Chronic inflammation strongly influences hematopoietic stem cell fate, biasing differentiation toward the myeloid lineage and altering cellular metabolism. Under inflammatory conditions, HSCs shift from glycolytic to oxidative metabolism, making mitochondrial quality control and mitophagy essential for maintaining stem cell function (Vannini et al., 2016). Decline in adaptive immunity becomes evident at the organismal level through reduced vaccine efficacy (Mittelbrunn and Kroemer, 2021; Li et al., 2023).

Aging cells, similarly to cancer cells, frequently upregulate inhibitory surface molecules. IL-6 signaling through STAT3 promotes survival and enhances PD-L1 expression, contributing to immune evasion (Onorati et al., 2022). Senescent cells further impair macrophage-mediated clearance by expressing CD47 and CD24 “do not eat me” signals, thereby dominating the local microenvironment and inhibiting efferocytosis (Schloesser et al., 2023). Distinct senescent subpopulations may also emerge: p21-high cells often represent an early protective response, whereas p16-high cells represent more permanent, irreversible arrest. Modulation of these pathways influences whether damaged cells undergo apoptosis or persist within tissues (Yosef et al., 2017; Majewska et al., 2024; Belmonte-Fernández et al., 2025).

Thymic involution represents one of the most visible features of immune aging. The diversity and plasticity of the T cell repertoire decline substantially, with a young individual harboring over 100 million unique TCRβ sequences compared with approximately 20–50 million in older individuals (Qi et al., 2014; Johnson et al., 2012). Thymic atrophy is a regulated process influenced by sex hormones, inflammatory signals, and metabolic status. For example, IL-6 can inhibit thymic function, whereas leptin and nutrient availability modulate lymphocyte development through mTOR-dependent mechanisms (Gruver and Sempowski, 2008; Howard et al., 1999; Mannick et al., 2018).

Generally, thymic involution is not irreversible. Experimental strategies such as transient suppression of the gonadal axis, transplantation of thymic tissue, or restoration of FOXN1 expression demonstrate partial regeneration of thymopoiesis and better immune response in aged models (Hince et al., 2008; Zhu et al., 2007; Oh et al., 2020). These observations highlight the degree to which immune aging reflects regulated remodeling rather than purely stochastic decline.

The gut microbiota also drives immune regulation across the lifespan. Short-chain fatty acids such as butyrate, produced through microbial fermentation of dietary fiber, promote differentiation of regulatory T cells and modulate systemic inflammation (Furusawa et al., 2013). As a histone deacetylase inhibitor, butyrate influences gene expression programs including FOXP3 and p21, potentially linking microbial metabolism with epigenetic control of immune function (Davie, 2003). Chronic dysbiosis, conversely, is associated with autoimmune disorders and persistent inflammatory activation (Coccia et al., 2024; Saccon et al., 2021).

Overall, immunological aging cannot be understood solely as functional decline. Rather, it reflects a progressive loss of regulatory flexibility within immune networks, leading to simultaneous immune insufficiency and chronic activation. This imbalance drives the persistence of dysfunctional cells, maintained inflammatory signaling, and the destabilization of tissue homeostasis that characterizes advanced aging.

Progressive immune dysregulation not only impairs the clearance of dysfunctional cells while remodeling the signaling environment within tissues. Among the pathways particularly sensitive to inflammatory and metabolic context is Wnt signaling, which combines regional and systemic cues to regulate cellular plasticity.

## Wnt signaling and epigenetic plasticity

In the following sections, Wnt signaling and TET enzymes are considered as an illustrative axis of metabolic–epigenetic control of plasticity, rather than as a universal module underlying all aspects of aging. If aging reflects a gradual decline in cellular plasticity, then key regulatory pathways controlling stem cell identity and tissue renewal become crucial to its interpretation. Among these, the Wnt signaling pathway and the β-catenin destruction complex appear to be particularly sensitive integrators of metabolic, epigenetic, and mechanical stimuli. Their activity influences not only developmental processes but also the ability of adult tissues to maintain regenerative flexibility throughout life, particularly under conditions such as physiological hypoxia, which protects stem cell potential.

### Hypoxia–Wnt–TET coupling in plasticity programs

Under hypoxic conditions, stem cells activate programs that promote self-renewal and preserve an immature state. HIF-1α plays a key role, forming a complex with β-catenin and inducing pluripotency-associated genes such as OCT4 and NANOG, alongside metabolic response genes including VEGF, GLUT1, and LDHA. Stem cells preferentially rely on glycolysis, which limits ROS production and supports a distinct, more “plastic” epigenetic state. A similar logic operates in cancer, where physiological hypoxia promotes survival and therapy resistance. HIF-1α can stabilize β-catenin-among others by downregulating APC-and together they activate antiapoptotic programs (e.g., BCL-2, survivin) and EMT-associated factors such as TWIST, SNAIL, and ZEB1 (Lee et al., 2012; Zhang et al., 2023).

Hypoxia also intersects directly with the epigenetic machinery. It alters TET1 function, which can act as a transcriptional coactivator for HIF-1. At the same time, the catalytic role of TET appears to persist even under hypoxia, suggesting that HIF-1 may redirect TET1 toward selected substrates. In induced hypoxia, hydroxymethylation increases and expression of Wnt-related genes rises, although TET enzymes also regulate this pathway under standard conditions (Kong et al., 2024). In this context, activation of Wnt signaling by TET1 has been implicated in gastric cancer (Meng et al., 2021).

Beyond stem cell niches, the Wnt pathway helps define tissue-level boundaries and responds to physical context. Its activity is influenced by contact inhibition and cytoskeletal tension through the Hippo pathway. Hippo signaling, via LATS1/2-mediated regulation of YAP/TAZ, is tightly coupled to canonical Wnt control: when Wnt is inactive, YAP/TAZ participate in the β-catenin destruction complex and reinforce repression; when Wnt is activated, this complex disassembles and YAP/TAZ can cooperate with β-catenin in transcriptional activation (Azzolin et al., 2014; Azzolin et al., 2012; Lee et al., 2018; Sharma et al., 2021; Zhao et al., 2010; Varelas et al., 2010). Crucially, the β-catenin destruction complex emerges here as an underappreciated integrating node where mechanical inputs, metabolic state, and proliferative cues converge.

With age, oxidative stress and altered growth signaling reshape Wnt output in a context-dependent manner. FOXO transcription factors, increasingly engaged under stress conditions, can antagonize Wnt/β-catenin signaling by competing with TCF/LEF complexes for β-catenin and redirecting transcription toward stress-response programs (Iyer et al., 2013). Yet FOX effects are not uniform-FOXD elements that boost Wnt signaling whereas other FOX subfamilies inhibit it-highlighting the strong contextual dependence of this regulatory layer (Moparthi and Koch, 2023). Growth signals counteract FOXO engagement through AKT-mediated FOXO inactivation and can simultaneously promote β-catenin nuclear accumulation (e.g., via phosphorylation at Ser552), shifting the balance toward TCF/LEF-driven transcription (Fang et al., 2007).

### TET/miRNA constraints and context-dependent Wnt output

Deficiency of TET enzyme function correlates with aging and is frequently oncogenic. Excessive methylation suppresses genes antagonistic to Wnt signaling, enabling aberrant pathway activation and, under permissive conditions, carcinogenesis (Duan et al., 2017; Xu et al., 2022). For example, DACT2 and SFRP2 contribute to the suppressive role of TET1 in nasopharyngeal cancer (Fan et al., 2018), while TET1 can inhibit pancreatic tumor proliferation and metastasis (Wu et al., 2019). Reduced Tet1/Tet2 expression has also been linked to hypermethylation of the DKK-1 promoter (Yu et al., 2019). Together with TET’s role in physiological hypoxia, this suggests that maintaining TET function is fundamentally important for normal stem cell regulation.

Essentially, TET loss does not produce a simple progeroid phenotype, consistent with its regulatory role and partial redundancy through passive and repair-associated demethylation. Nonetheless, Tet1 deficiency in mice produces marked developmental and proliferative defects in selected tissues, including reduced proliferation in the intestinal epithelium (Kim et al., 2016). In pluripotent contexts, TET1 supports proliferation and identity programs. It can be recruited by Nanog and targeted to enhancers and promoters of key genes such as Oct4, while its expression is itself regulated by core pluripotency factors (Oct4, Sox2) and reinforced through autoregulatory binding (Chen et al., 2015; Costa et al., 2013; Olariu et al., 2016). Tet enzymes have also been implicated in telomere regulation in mouse embryonic stem cells (Yang et al., 2016). More broadly, TET1 binds preferentially to promoter regions of many housekeeping genes, even when methylation is not high, consistent with a subtler role in maintaining local epigenetic status and preventing inappropriate *de novo* methylation (Williams et al., 2011; Neri et al., 2013; Ooi et al., 2007; Wu et al., 2011). It would, however, be overly simplistic to interpret reduced TET activity as uniformly detrimental. In selected stem cell compartments, partial attenuation of TET function has been associated with increased proliferative output or competitive advantage. Yet such gains appear to come at a cost: enhanced clonal bias, reduced lineage flexibility, and diminished long-term regulatory stability (Hong T. et al., 2023). From the perspective proposed here, these observations do not contradict the plasticity framework but rather illustrate a shift toward more constrained and less reversible network states.

MicroRNA networks add another layer of stabilization and, potentially, rigidity. miR-29 directly inhibits enzymes responsible for active DNA demethylation (TET1/2/3) and *de novo* DNA methylation (DNMT3A/3B) (Hysolli et al., 2016), effectively limiting both demethylation intermediates and new methylation site formation. In this way, miR-29 can “freeze” existing methylation patterns, impair somatic cell reprogramming, and stabilize differentiation (Fráguas et al., 2017). Notably, miR-29 also interacts with Wnt signaling. During osteoblast differentiation from human mesenchymal stem cells, miR-29a suppresses pathway inhibitors, activating Wnt/β-catenin and inducing targets such as c-MYC, while also promoting its own expression in a positive feedback loop (Kapinas et al., 2009). In mouse neurogenesis, miR-29 can activate Wnt signaling by inhibiting Icat (an inhibitor of β-catenin/TCF function) (Shin et al., 2014), and miR-29b can directly suppress GSK3-β via its 3′UTR (Fráguas et al., 2017; Liu et al., 2011).

Collectively, these mechanisms are consistent with the idea that aging involves a gradual loss of regulatory flexibility rather than a simple accumulation of damage. Wnt signaling and TET-dependent epigenetic control are developed as one illustrative axis of metabolic–epigenetic regulation of cellular plasticity, rather than as a uniquely central module. Other pathways, including Notch, TGF-β, NF-κB, the p53/p21/p16 network, YAP/TAZ and several chromatin-remodeling complexes, have been extensively characterized in apoptosis, senescence and tumorigenesis and clearly constrain or sustain plasticity in tissue-specific ways (Hoare et al., 2016; Ito et al., 2017; Iyer et al., 2013; Azzolin et al., 2012; Azzolin et al., 2014; Ito et al., 2018; Sharma et al., 2021). Integrating these diverse regulators into a common framework of regulatory plasticity and rigidity remains an important task for future work.

## Restoring regulatory plasticity: therapeutic perspectives

If aging reflects a progressive restriction of regulatory plasticity, therapeutic approaches may differ substantially into those that remove dysfunctional cells and those that attempt to restore or modulate regulatory networks.

Senolytics aim to eliminate senescent cells from tissues already exhibiting pathological features. Various strategies under investigation, including prodrugs activated by lysosomal β-galactosidase (Cai et al., 2020). A widely studied combination is dasatinib plus quercetin. Together, they improve physical performance, osteogenesis, and muscle regeneration in aged mice and have been tested in idiopathic pulmonary fibrosis (Krzystyniak et al., 2022; Zhou et al., 2021; Dungan et al., 2022; Justice et al., 2019). Inhibitors of Bcl-2 family proteins, including Bcl-xL antagonists, show additional potential (Zhu et al., 2016; Jiang et al., 2024; Saleh et al., 2020). Immunological strategies-such as antibody–drug conjugates or CAR-T cells targeting senescence-associated surface markers like uPAR-offer another route, although the lack of a unique senescence-specific antigen remains a major limitation (Poblocka et al., 2021; Amor et al., 2020; Eskiocak et al., 2025; Amor et al., 2024).

Senomodulators seek to modulate cellular biochemistry rather than eliminate cells. Compounds such as rapamycin, metformin, and resveratrol influence central metabolic regulators. Metformin extends lifespan in experimental models when administered early (Anisimov et al., 2011), while resveratrol improves healthspan across species (Baur et al., 2006; Bass et al., 2007; Pallauf et al., 2016). Although its direct activation of SIRT1 remains debated, its capacity to stimulate AMPK and modulate metabolic stress responses is well supported (Park et al., 2012; Lan et al., 2017; Price et al., 2012). More broadly, dietary polyphenols appear to exert cumulative advantages, especially in combination (e.g., quercetin slowing resveratrol metabolism and enhancing IIS and NF-κB modulation). Several small molecules influence alternative splicing and may partially reverse age-associated transcriptomic changes (Xiao et al., 2023; Latorre et al., 2017).

Other approaches attempt to limit epigenetic drift or directly modulate chromatin regulators, including histone deacetylase inhibitors. Butyrate, a microbiota-derived short-chain fatty acid, functions as a class I/II HDAC inhibitor and extends lifespan in invertebrate models (McIntyre et al., 2019), linking metabolic inputs to chromatin control.

The regulatory role of TET enzymes points to additional therapeutic avenues. Vitamin C enhances TET activity and has been shown to restore altered TET expression in inflammatory contexts (Li F. et al., 2020; Liu et al., 2020). Loss of Tet2/3 in Treg cells induces severe inflammation (Yue et al., 2019), highlighting the importance of active DNA demethylation in immune balance. Experimental data further suggest that TET enzymes may reduce transcriptional noise (Kang et al., 2015). Vitamin D receptor (VDR) signaling intersects with TET2, Wnt, mTOR, and SIRT1 pathways (Català-Moll et al., 2022; Al-Hendy et al., 2016; Ferrer-Mayorga et al., 2019; Sari et al., 2020), illustrating how micronutrients also shape wider regulatory networks rather than single molecular targets.

The relationship between SIRT1 and Wnt signaling highlights the contextual nature of regulatory modulation. SIRT1 can deacetylate β-catenin and influence its nuclear localization, with effects that vary depending on metabolic state, cell type, and pathological context (Bartoli-Leonard et al., 2018; Wei et al., 2015; Chocarro-Calvo et al., 2013; García-Jiménez et al., 2014; Hu et al., 2020; Chen X. et al., 2019). Similarly, interventions targeting components of the Wnt pathway–such as tankyrase or porcupine inhibitors–hold promise in oncology but may be unsuitable for systemic anti-aging strategies due to the pathway’s fundamental role in tissue homeostasis (Kim, 2018; Lung et al., 2024).

Finally, microRNA-based interventions illustrate both potential and risk. miR-29, a pleiotropic regulator of DNA methylation and Wnt signaling, can be targeted using antisense oligonucleotides. Such approaches may improve metabolic, neurodegenerative, or skeletal parameters (Hu et al., 2014; Kurtz et al., 2015; Mei et al., 2024; Ding et al., 2024). However, because miR-29 drives the stabilization of differentiation programs, its manipulation must be considered in the context of tissue-specific regulatory needs.

Taken together, these examples are intended less as a ranked list of primary anti-aging targets than as proof-of-concept interventions showing that regulatory plasticity can, in principle, be modulated at multiple levels, including senescent cell burden, metabolic signaling, chromatin regulation, and microRNA-dependent stabilization of cell states (Cai et al., 2020; Zhou et al., 2021; Wu et al., 2018; Hysolli et al., 2016). In this sense, pathways involving TET enzymes, Wnt signaling, sirtuins, or miR-29 should be viewed as illustrative entry points into a broader regulatory landscape rather than as uniquely privileged modules. At the same time, attempts to reopen plasticity are unlikely to be uniformly beneficial. Excessive or misdirected plasticity may facilitate dedifferentiation, clonal expansion, fibrosis, or tumorigenesis, particularly in tissues already burdened by oncogenic mutations or chronic inflammatory remodeling (Lehmann et al., 2020; Liu et al., 2007; Chen X. et al., 2019; Yang et al., 2023). Conversely, further restricting plasticity may in some settings protect tissue identity or suppress malignant transformation, albeit at the cost of regenerative potential (Demaria et al., 2014; Ito et al., 2017; Hoare et al., 2016). The therapeutic challenge is therefore not simply to increase plasticity, but to modulate it within a controlled range that preserves adaptive responsiveness without destabilizing cellular identity.

Gathered together, these strategies suggest that successful intervention is unlikely to depend on a single pathway. Rather than simply suppressing damage or inflammation, future therapies may need to complement such approaches by modulating the dynamic balance between signaling networks, epigenetic regulation, and metabolic state in ways that transiently increase adaptive plasticity without destabilizing cellular identity. This framework does not imply that reopening plasticity is universally desirable; instead, it highlights the need to define where, when, and to what extent regulatory flexibility can be safely restored in specific tissues and disease contexts.

If aging is at least partly shaped by a progressive restriction of regulatory plasticity, this framework carries several operational implications and testable hypotheses. First, biological age may need to be assessed not only by static molecular damage or epigenetic marks, but by the dynamic capacity of regulatory networks to respond to transient perturbations and return to homeostasis. Second, interventions that transiently restore regulatory flexibility (even without substantially reducing accumulated molecular damage) may, in some contexts, delay functional decline more effectively than interventions acting solely through damage suppression.

Finally, experimental systems comparing cells or tissues of similar molecular age but differing adaptive responsiveness may help test whether loss of regulatory plasticity represents a primary driver of aging, an amplifier, an adaptive response that later becomes maladaptive, or mainly a downstream consequence of molecular damage and tissue stress.

## Concluding perspectives

Aging is usually described as the progressive accumulation of molecular damage, but the evidence discussed here suggests that this view is incomplete. In many contexts, regulatory systems are tuned to preserve stability first: damage is walled off by scarring, transformation risk is contained by senescence, and stem cells gradually lose the capacity to reset identity and rebuild tissues. Mechanisms that protect development and tissue architecture can therefore, over time, reduce how flexibly cells respond to stress and injury.

From this perspective, aging can be viewed as a gradual narrowing of epigenetic and regulatory plasticity. Changes in chromatin organization, DNA methylation dynamics, transcriptional fidelity, mitochondrial–nuclear communication, and immune surveillance together influence how wide the accessible regulatory state space remains in a tissue. Classical hallmarks such as genomic instability, mitochondrial dysfunction, proteostatic stress, epigenetic drift, senescence, and immunoaging can therefore be reinterpreted not simply as isolated causes, but as processes that constrain this state space in partially overlapping ways.

This perspective is not intended to replace existing aging models, nor does it establish loss of regulatory plasticity as a primary driver of aging in all contexts. Rather, it provides an integrative lens through which diverse mechanisms can be examined within a common framework and generates testable hypotheses about how aging systems lose adaptive responsiveness. In this view, one key question is not only how much molecular damage accumulates, but also how effectively regulatory networks can respond to perturbation, recover, and return to adaptive states rather than becoming fixed in maladaptive ones. In this sense, the framework does not simply relabel existing hallmarks, but shifts attention toward the reversibility, responsiveness, and coordination of regulatory states as features that may differ even among tissues with comparable burdens of molecular damage.

A practical way to begin operationalizing regulatory plasticity would be to compare how cells of different ages and differentiation states respond to and recover from standardized, transient stresses such as heat shock or hypoxia. In such an experimental design, stem-like, differentiated and senescent cells would be exposed to the same short-term perturbation, and the subsequent recovery of transcriptional programs, chromatin accessibility and DNA methylation at selected loci would be monitored over time. The speed and completeness of this recovery could serve as an integrated readout of the dynamic capacity of regulatory networks. Such perturbation-response designs would allow testing whether aged or senescent cells occupy states that are less capable of reversible reconfiguration despite comparable levels of underlying molecular damage.

This framework also has practical implications. Biological age may need to be assessed not only with static markers of damage or epigenetic clocks, but also with dynamic measurements of how regulatory networks respond to defined challenges and return to baseline. Likewise, interventions that modulate plasticity should not be understood as attempts to maximize flexibility indiscriminately. In some settings, restricted plasticity protects tissue identity and suppresses malignant transformation; in others, it contributes to regenerative failure, chronic inflammation, or stable senescence-associated dysfunction.

The central challenge for future work is therefore to determine when reduced plasticity is adaptive, when it becomes maladaptive, and how far regulatory flexibility can be safely restored at the level of chromatin, metabolism, and signaling without destabilizing cellular identity. It will also be essential to clarify whether loss of plasticity acts primarily as a driver, amplifier, adaptive response, or downstream consequence of aging-related damage, thereby defining how this framework can be translated into experimentally grounded and physiologically coherent intervention strategies.
